# Exosomes Derived From Adipose-Derived Mesenchymal Stem Cells Ameliorate Radiation-Induced Brain Injury by Activating the SIRT1 Pathway

**DOI:** 10.3389/fcell.2021.693782

**Published:** 2021-07-29

**Authors:** Mengdong Liu, Yunshu Yang, Bin Zhao, Yuefan Yang, Jing Wang, Kuo Shen, Xuekang Yang, Dahai Hu, Guoxu Zheng, Juntao Han

**Affiliations:** ^1^Department of Burns and Cutaneous Surgery, Xijing Hospital, Air Force Military Medical University, Xi’an, China; ^2^Department of Biomedical Engineering, Air Force Military Medical University, Xi’an, China; ^3^Department of Neurosurgery, Xijing Hospital, Air Force Military Medical University, Xi’an, China; ^4^State key laboratory of Cancer Biology, Department of Immunology, Air Force Military Medical University, Xi’an, China

**Keywords:** radiation, brain injury, exosomes, mesenchymal stem cells, oxidative stress

## Abstract

**Objective:**

Studies have shown that the therapeutic effects of mesenchymal stem cells (MSCs) are mediated in a paracrine manner, mainly through extracellular vesicles such as exosomes. Here, we designed a study to investigate whether exosomes derived from adipose-derived mesenchymal stem cells (ADMSC-Exos) had protective effects in a rat model of radiation-induced brain injury and in microglia.

**Methods:**

Male adult Sprague-Dawley (SD) rats were randomly divided into three groups: the control group, the radiation group (30 Gy), and the radiation + exosomes group (30 Gy + 100 ug exosomes). Meanwhile, microglia were divided into four groups: the control group, the radiation group (10 Gy), the radiation + exosomes group (10 Gy + 4 ug exosomes), and radiation + exosomes + EX527 group (10 Gy + 4 ug exosomes + 100 nM EX527). Tissue samples and the levels of oxidative stress and inflammatory factors in each group were compared.

**Results:**

Statistical analysis showed that after irradiation, ADMSC-Exos intervention *in vivo* significantly reduced the levels of caspase-3, malondialdehyde (MDA), 8-hydroxydeoxyguanosine (8-OHdG), tumor necrosis factor-α (TNF-α), interleukin-4 (IL-4), and promoted the recovery of superoxide dismutase (SOD), catalase (CAT), IL-4, and IL-10. Moreover, ADMSC-Exos intervention inhibited microglial infiltration and promoted the expression of SIRT1. Furthermore, the results *in vitro* showed that the above effects of ADMSC-Exos could be reversed by SIRT-1 inhibitor EX527.

**Conclusion:**

This study demonstrated that ADMSC-Exos exerted protective effects against radiation-induced brain injury by reducing oxidative stress, inflammation and microglial infiltration via activating the SIRT1 pathway. ADMSC-Exos may serve as a promising therapeutic tool for radiation-induced brain injury.

## Introduction

Radiotherapy plays an important role in the treatment of brain tumors, head and neck cancers, and arteriovenous malformations (Na et al., 2014). However, radiotherapy not only causes tumor cell necrosis but also affects the surrounding healthy tissue, and patients who receive radiotherapy are at risk of developing radiation-induced brain injury (RIBI) (Rahmathulla et al., 2013). The process of radiation-induced brain injury is a complicated, cascaded and dynamic process, and several theories have been proposed to explain the development of radiation-induced brain injury, including direct injury from radiation (Oh et al., 2013), damage to the cerebrovascular system (Kamiryo et al., 2001), immunoinflammatory responses (Lumniczky et al., 2017; Bostrom et al., 2018), and oxidative stress (Liao et al., 2017). In recent years, several preclinical studies have demonstrated that interventions such as modulating inflammation and reducing oxidative stress injury can prevent or ameliorate radiation-induced brain injury. One of those interventions is mesenchymal stem cell (MSC) therapy (Balentova and Adamkov, 2015).

Mesenchymal stem cells have emerged as promising therapeutic measures for different clinical applications, including tissue engineering, autoimmune diseases, and graft-versus-host diseases (Kiang, 2016; Drommelschmidt et al., 2017). Previous studies have demonstrated that MSCs could protect against radiation-induced liver injury (Francois et al., 2013), promote wound healing in radiation-induced skin injury (Xie et al., 2013; Sun et al., 2019), improve survival and mitigate gastrointestinal syndrome in irradiated mice (Hu et al., 2010; Kiang, 2016). However, in recent years, an increasing number of studies have shown that the therapeutic effects of MSCs are mediated in a paracrine manner, mainly through extracellular vesicles such as exosomes (Drommelschmidt et al., 2017; Giebel et al., 2017). Exosomes are small biological lipid membrane vesicles with diameters of 40–100 nm (Trams et al., 1981). They serve as vital extracellular communicators by transporting their contents, such as microRNAs (miRNAs), messenger RNAs (mRNAs), cell adhesion molecules, cytokines and proteins, to target recipient cells (Ren, 2018). Whether exosomes from MSCs can alleviate radiation-induced brain injury is still unclear.

Thus, we designed this study to investigate whether exosomes derived from adipose-derived MSCs (ADMSC-Exos) had neuroprotective effects on the injured brain tissues of irradiated animals.

## Materials and Methods

### Animals

Adult male Sprague-Dawley (SD) rats weighing between 250 and 300 g were provided by the Experimental Animal Center of the Air Force Military Medical University. All rats were housed five per cage under a constant 12-h light/dark cycle at room temperature. Food and water were available *ad libitum*. All experimental protocols and animal handling procedures were performed in accordance with the National Institutes of Health (NIH) guidelines for the use of experimental animals and were approved by the Institutional Animal Care and Use Committee of the Air Force Military Medical University.

### Irradiation

Irradiation was performed using a ^60^Co gamma-ray facility (Radiation Center of Air Force Military Medical University, Xi’an, China) at a dose rate of 1.59 Gy/min and a total dose of 30 Gy. Animals were placed in perforated plastic bottles before receiving a single dose of 30 Gy by stereotactic irradiation (at a dose rate of 1.59 Gy/min and a distance of 65 cm from the source). The animals’ movements were restricted by anaesthetization. The head of each rat was placed in the exposure field (5 × 2.5 cm^2^) which covered the whole brain from the postaurem line to the post canthus line.

### Cell Culture

#### Adipose-Derived Mesenchymal Stem Cells Harvest and Culture

Adult male SD rats weighing between 250 and 300 g were anesthetized by isoflurane inhalation, and subcutaneous adipose tissue in the groin was carefully dissected. Afterward, the animals were sacrificed. The adipose tissue was minced and washed with phosphate-buffered saline (PBS) twice and then digested with collagenase type I (CLS I; 2 mg/ml; Gibco, Grand Island, United States) for 60 min at 37°C with shaking. After centrifugation for 10 min at 620 × *g* and the removal of the supernatant, the precipitate was resuspended in ADMSCs culture medium consisting of DMEM/F12 (Gibco, Life Technologies, Carlsbad, CA, United States) containing 100 U/ml penicillin, 100 mg/ml streptomycin (Gibco BRL, Grand Island, NY, United States), 0.2 mM L-ascorbic acid-2-phosphate (A2P; Sigma, St. Louis, MO, United States), and 10% fetal bovine serum (Excell Bio, Shanghai, China). The cells were cultured at 37°C in a humidified incubator with 5% CO_2_. For characterization of ADMSCs differentiation, Oil-Red-O, Alizarin red, and Alcian blue staining were conducted to identify adipogenic, osteogenic, and chondrogenic differentiation, respectively. After three passages, ADMSCs were cultured in adipogenesis-, osteogenesis-, or chondrogenesis-inducing medium for 2 weeks. Then, the cells were stained with Oil-Red-O, Alizarin red, and Alcian blue.

#### Rat Microglia Culture

Primary microglia from SD rats were obtained from the Neurological Lab of the Tangdu Hospital in China. Microglia were maintained in Dulbecco’s Modified Eagle’s Medium containing 5% fetal bovine serum (Excell Bio, Shanghai, China), 100 mg/l streptomycin and 100 units/ml penicillin (Gibco BRL, Grand Island, NY, United States) in a humidified 5% CO_2_, 95% air atmosphere. Microglia were seeded into 24-well plates at 1 × 10^5^ cells/well 48 h before irradiation.

#### Isolation and Characterization of Exosomes

Third-generation ADMSCs were cultured in DMEM/F12 supplemented with 10% exosome-depleted FBS for 48 h, and the culture supernatants were carefully collected. For exosome isolation, the cells and cell debris in the supernatants were removed by centrifugation at 3,000 × *g* for 15 min. Then, the exosomes were isolated from culture supernatants of ADMSCs using a total exosome isolation kit (Invitrogen, Asheville, NC, United States) according to the manufacturer’s instructions. Isolated exosomes were suspended in PBS and stored at −80°C. The protein content of exosome suspension was quantified using the bicinchoninic acid (BCA) protein assay kit (Beyotime, Shanghai, China). The morphology of isolated exosome was analyzed by transmission electron microscopy (TEM), and the distribution of size was analyzed by nanoparticle tracking analysis (NTA; Zeta View system). We further characterized exosomes by western blot analysis of the exosomal markers CD9, CD63, and CD81 (Abcam, Cambridge, United Kingdom).

### Experimental Design

(i) Investigate whether ADMSC-Exos treatment can alleviate radiation-induced brain injury. Animals were randomly assigned to three groups as follows: (1) control group (Ctr group): SD rats were injected with 200 μl of PBS via the tail vein; (2) radiation group (RT group): SD rats were injected with 200 μl of PBS via the tail vein 15 min before irradiation; and (3) radiation + exosomes group (RT + Exo group): SD rats were injected with 100 μg of exosomes (suspended in PBS to a final volume of 200 μl) via the tail vein 15 min before irradiation. Both the RT group and RT + Exo group were irradiated with 30 Gy. Twenty-five rats per group were used in this study.

(ii) Evaluate the influences of ADMSC-Exos on the irradiated microglia and explore the role of SIRT-1 in this process. Firstly, microglia were randomly divided into three groups: (1) control group (Ctr group): cells were treated with 10 μl PBS; (2) radiation group (RT group): cells were treated with 10 μl PBS 15 min before irradiation; (3) radiation + exosomes group (Exo group): cells were preincubated with exosomes (2.0, 4.0, or 8.0 μg suspended in PBS to a final volume of 10 μl) 15 min before irradiation. Cells in the RT and Exo groups were irradiated with a single dose of 10 Gy at a rate of 3 MeV/min. The cell samples and culture supernatants were collected at 24 h after irradiation, oxidative stress and inflammatory factors were detected. Based on the results of the first step, the second step was carried out. Microglia were randomly divided into four groups: (1) control group (Ctr group): cells were treated with 10 μl PBS; (2) radiation group (RT group): cells were treated with 10 μl PBS 15 min before irradiation; (3) radiation + exosomes group (Exo group): cells were preincubated with 4.0 μg exosomes (suspended in PBS to a final volume of 10 μl) 15 min before irradiation; (4) radiation + exosomes + EX527 group (EX527 group): cells were treated with 100 nM EX527 15 min before irradiation and other interventions were the same as Exo group. Cells in the RT, Exo and EX527 groups were irradiated with a single dose of 10 Gy at a rate of 3 MeV/min. The cell samples and culture supernatants were collected at 24 h after irradiation, the expressions of CD68, SIRT1, P65, oxidative stress markers, and inflammatory factors were detected.

### Western Blot Analysis

Protein samples (5 μg/lane) from exosomes, hippocampus, and microglia were loaded onto polyacrylamide gels, separated by electrophoresis, and then transferred to polyvinylidene difluoride membranes (PVDF). The membranes were blocked with 5% non-fat milk at room temperature for 3 h and incubated with rabbit monoclonal anti-rat CD63 (1:500, Abcam, Cambridge, United Kingdom), CD9 (1:500, Abcam, Cambridge, United Kingdom), CD81 (1:500, Abcam, Cambridge, United Kingdom), cleaved Caspase 3 (1:1,000, Cell Signaling Technology, Boston, United States),SIRT1 (1:1,000, Abcam, Cambridge, United Kingdom), CD68 antibodies (1:500, Abcam, Cambridge, United Kingdom), Ac-p65 (1:1,000, Cell Signaling Technology, Boston, United States) and anti-p65 (1:1,000, Cell Signaling Technology, Boston, United States) antibodies overnight at 4°C, followed by incubation with horseradish peroxidase-conjugated goat anti-rabbit secondary antibodies (1:3,000, Boster, Wuhan, China). Proteins were visualized by an enhanced chemiluminescence system (Alpha Innotech, United States).

### Flow Cytometry

Adipose-derived mesenchymal stem cells at passages 3–5 were harvested. Approximately 1 × 10^5^ ADMSCs were fixed in neutralized 2% paraformaldehyde (PFA) solution for 30 min, washed twice with PBS, and labeled with fluorescence-conjugated antibodies against CD29, CD31, CD44, and HLA-DR (BD Biosciences) at room temperature for 30 min. The primary antibodies were directly conjugated with FITC and phycoerythrin. Non-specific FITC-conjugated IgG was used to stain the cells as a control. The cells were then analyzed by a FACS Aria III (Becton-Dickinson, San Jose, CA, United States).

Fresh hippocampal tissues were harvested at various time points (0 h, 24 h, 3 days, and 7 days) from three rats in each group, and single-cell suspensions were prepared as described (Seibenhener and Wooten, 2012). For apoptosis analysis, cells were washed thrice with PBS. Then, cells were incubated with 100 μL of RNase (100 mg/L) for 30 min at 37°C and then stained with PI and annexin V-FITC at 4°C for 30 min. The cell cycle and apoptosis were monitored at 488 nm. Each sample containing 1 × 10^6^ cells.

### Histopathological Examination

The animals were sacrificed by cervical dislocation preirradiation (0 h) and at 24 h, 3 days, and 7 days after irradiation. Parts of sacrificed rats were initially perfused with 10% formalin. After fixation, a craniotomy was performed. Then, the harvested brain tissues were fixed with 10% formalin, dehydrated by graded ethanol, and embedded in paraffin. Five-micrometer-thick slices were cut for Nissl staining. The neuronal density was determined by the average number of surviving hippocampal Cornu Ammonis (CA) 1 in each 1-mm section, with three sections of bilateral hippocampal slices.

### Measurement of Caspase-3 Activity

Hippocampal tissue was harvested at various time points (0 h, 24 h, 3 d, and 7 d), dissected, homogenized in chilled PBS (0.1 M, pH 7.4), and then centrifuged at 10,000 × *g* at 4°C for 10 min. The supernatants were collected, aliquoted, and stored at −80°C for subsequent analysis. The tissue protein concentration was determined by a BCA protein assay kit (Beyotime, Shanghai, China). Caspase-3 activity in the hippocampus was determined by using a Caspase-3/CPP32 colorimetric assay kit (Beyotime, Shanghai, China) according to the manufacturer’s instructions.

### Measurement of Antioxidant Enzymes

The activities of superoxide dismutase (SOD) and catalase (CAT) in the hippocampus and microglia were determined by using commercial kits (Cayman Chemical Company, Ann Arbor, MI, United States) according to the manufacturer’s instructions.

### Measurement of Oxidative Products

The homogenates obtained as previously described were also used to measure the levels of malondialdehyde (MDA) and 8-hydroxydeoxyguanosine (8-OHdG). The levels of 8-OHdG and MDA were measured by commercial kits (Genox Corporation, Baltimore, MD, United States) according to the manufacturer’s instructions.

### Measurement of NO/Nitrite and Inflammatory Cytokines

NO production was indirectly assessed by measuring the nitrite levels in the culture supernatants by a commercial kit (Beyotime, Shanghai, China). The levels of tumor necrosis factor-α (TNF-α), interleukin-1β (IL-1β), interleukin-4 (IL-4), and interleukin-10 (IL-10) in the hippocampus and the supernatant of microglia were measured by enzyme-linked immunosorbent assay (ELISA) kits (Beyotime, Shanghai, China) according to the manufacturer’s instructions.

### Immunohistochemistry

Immunohistochemistry was performed to detect changes of microglia in the hippocampus at 3 days after irradiation. Paraffin sections were initially soaked in xylene, deparaffinized by graded ethanol, permeabilized with 3% Triton X-100 for 10 min, blocked with 10% normal donkey serum in PBS for 60 min at room temperature and incubated overnight with primary antibodies at 4°C. Microglia were detected with rabbit anti-rat IBA-1 antibodies (1:500, Wako Pure Chemical Industries, Osaka, Japan). Primary antibodies were visualized by using Alexa Fluor 594-conjugated donkey anti-rabbit secondary antibodies (1:500, Molecular Probes, Eugene, OR, United States). After nuclear staining with DAPI, the sections were analyzed on a Nikon fluorescence microscope. The number of IBA1-positive cells were counted in six standardized fields. The specific steps were the same as we described before (Liu et al., 2015).

Immunohistochemistry was also performed to detect the expression of CD68 in Microglia at 24 h after irradiation. Microglia from 24-well were fixed with 4% PFA for 30 min, permeabilized with 0.1% Triton X-100 for 30 min, and then blocked with 1% BSA for 1 60 min. Thereafter, the cells were incubated with CD68 antibodies (1:200, Abcam, Cambridge, United Kingdom) and IBA antibodies (1:200, Molecular Probes, Eugene, OR, United States) overnight at 4°C. Then, cells were incubated with Alexa Fluor 594-conjugated donkey anti-rabbit secondary antibody (1:500, Molecular Probes, Eugene, OR, United States) at 37°C for 60 min and DAPI (10 μg/mL) for 5 min. Florescence images were acquired and processed by a Nikon fluorescence microscope.

### Statistical Analysis

All statistical analyses were performed using SPSS 19.0, a statistical software package. The data generated are expressed as means ± SD. Statistical evaluation of the data was performed by one-way analysis of variance (ANOVA). A value of *p* < 0.05 was considered statistically significant.

## Results

After irradiation, all rats survived and showed normal daily activities, including feeding and drinking. No paralysis and convulsion were observed, and no significant difference was found in body weight gain among those groups.

### Characterization of ADMSC-Exos

**FIGURE 1:**
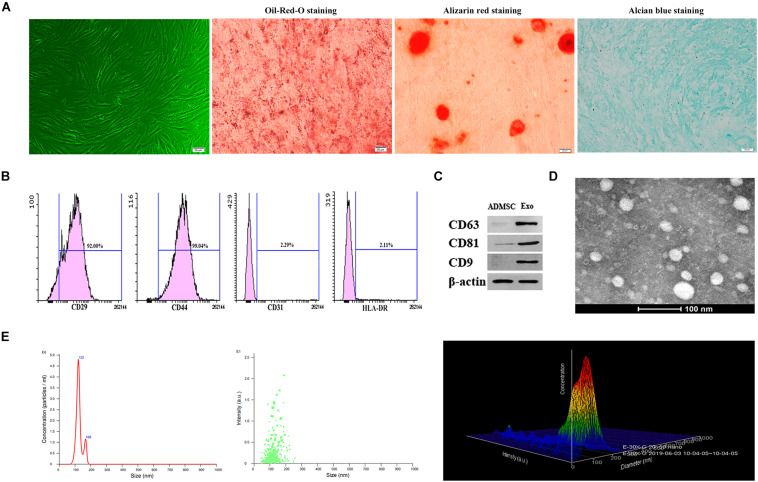
Characterization of ADMSCs and authentication of ADMSC-derived exosomes (ADMSCs-Exos). **(A)** ADMSCs morphology observed by inverted microscope. Adipogenic, osteogenic, and chondrogenic differentiations measured by Oil-Red-O staining, Alizarin red staining and Alcian blue staining (scale bar = 20 μm). **(B)** Flow cytometry analysis showed that these cells were highly positive for CD29 (92%) and CD44 (99.04%), but negative for CD31 (2.29%), and HLA-DR (2.11%). **(C)**. Exosome specific markers (CD63, CD81, CD9) were detected by Western blot. **(D)** The morphology of ADMSCs-Exos analyzed by transmission electron microscopy images. **(E)** The particle size distribution of ADMSCs-Exos detected by nanoparticle tracking analysis (NTA).

Under the microscope, ADMSCs presented as spindle-shaped and were aggregated in a swirled pattern. In addition, Oil-Red-O, Alizarin red, and Alcian blue staining showed that ADMSCs successfully differentiated into adipocytes, osteoblasts, and chondrocytes, respectively (Figure 1A). ADMSCs were identified by flow cytometry. A relatively high number of CD29- and CD44-positive cells and few CD31- and HLA-DR-positive cells were observed (Figure 1B). The exosome surface markers CD9, CD63, and CD81 were measured by western blotting, and the results showed that all three markers were highly expressed in the purified exosomes (Figure 1C). Exosomes appeared to be cup- or sphere-shaped particles as observed by TEM (Figure 1D). Exosomes isolated from 100 ml culture supernatants of ADMSCs were resuspended in 100 μl PBS, the results of BCA assay showed that the mean concentration of resuspended exosomes was 6.2 ug/ul. After diluting the resuspended exosomes 1000 times, NTA analysis was performed. NTA analysis identified the mean vesicle diameter was 126.5 nm and the concentration was 1.51 × 10^8^ particles/ml (Figure 1E). These results in all indicated that the nanoparticles were consistent with the defined exosomes.

### Adipose-Derived Mesenchymal Stem Cells Protected Hippocampal Cells From Radiation-Induced Apoptosis

**FIGURE 2:**
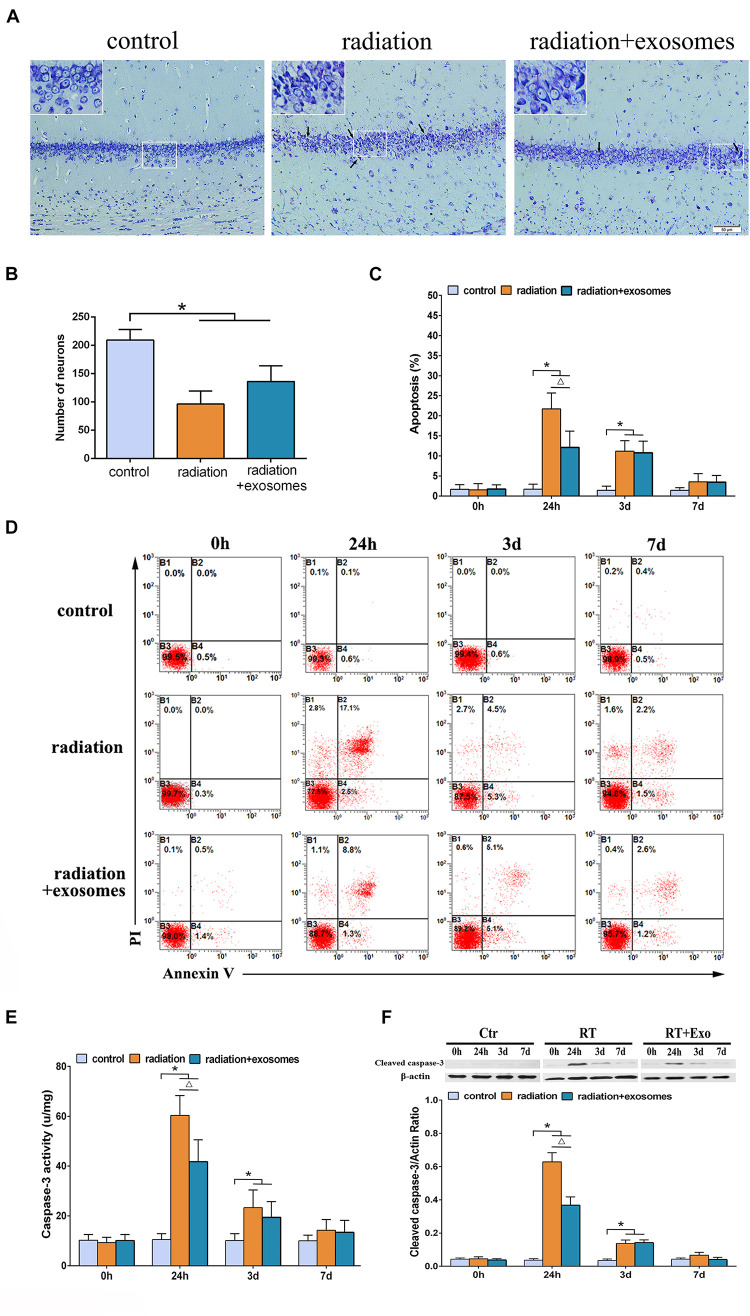
Adipose-derived mesenchymal stem cells protected hippocampal cells from radiation-induced apoptosis. **(A)** Nissl-stained sections of the hippocampus CA1 area at 3 days after irradiation. Chromatic agglutination and karyopyknosis were observed in both the RT and RT + Exo groups. Arrows indicate the karyopyknosis (scale bar = 50 μm). **(B)** Neuron count in the hipocampal CA1 region at 3 days after irradiation (*n* = 6). **(C)** Proportions of apoptotic cells in hippocampus determined by flow cytometry (*n* = 3). **(D)** Representative flow cytometry plots of Annexin V FITC and PI stained cells. **(E)** Changes of caspase-3 activity in the hippocampus after irradiation (*n* = 6). **(F)** Expression of cleaved caspase-3 in the hippocampus after irradiation determined by Western blot (*n* = 3) (*p < 0.05, compared with the Ctr; ^△^ *p* < 0.05, compared with the RT).

Under the optical microscope, Nissl-stained sections from the Ctr group showed hippocampal neurons with regular arrangements and clearly visible nuclei. At 3 days after irradiation, chromatic agglutination and karyopyknosis were observed in both the RT and RT + Exo groups (Figure 2A). The numbers of neurons were significantly decreased in the CA1 region in the RT group (96.3 ± 23.2/mm ^2^) and RT + Exo group (136.2 ± 27.8/mm ^2^) compared with those in the Ctr group (209.5 ± 18.8/mm ^2^) (*p* < 0.05). However, the numbers of neurons had no significant differences between RT group and RT + Exo group at 3 days after irradiation (Figure 2B). Flow cytometric analysis showed that the apoptosis rate in the Ctr group was significantly lower than that in the RT group or RT + Exo group at 24 h and 3 days after irradiation (*p* < 0.05). Compared with RT goup, ADMSC-Exos intervention significantly reduced the apoptosis rate in the RT + Exo group at 24 h after irradiation (*p* < 0.05). But no significant difference was found in apoptosis rate between the RT group and RT + Exo group at 3 days after irradiation (Figures 2C,D). Statistical analysis showed that the caspase-3 activities in the RT and RT + Exo groups was significantly higher than those in the Ctr group at 24 h and 3 days after irradiation (*p* < 0.05). At 24 h after irradiation, the caspase-3 activity in the RT group was significantly higher than that in the RT + Exo group (*p* < 0.05) (Figure 2E). Western blot analysis showed that at 24 h and 3 days after irradiation, the protein expression of cleaved caspase-3 in the RT + Exo and RT groups was significantly higher than that in the Ctr group (*p* < 0.05). At 24 h after irradiation, the protein expression of cleaved caspase-3 in the RT + Exo group was significantly lower than that in the RT group (*p* < 0.05) (Figure 2F). These results suggested that radiation could cause apoptosis of the hippocampal cells, and ADMSC-Exos intervention prevented radiation-induced apoptosis.

### Adipose-Derived Mesenchymal Stem Cells Alleviated Radiation-Induced Oxidative Stress and Inflammation in the Hippocampus

**FIGURE 3:**
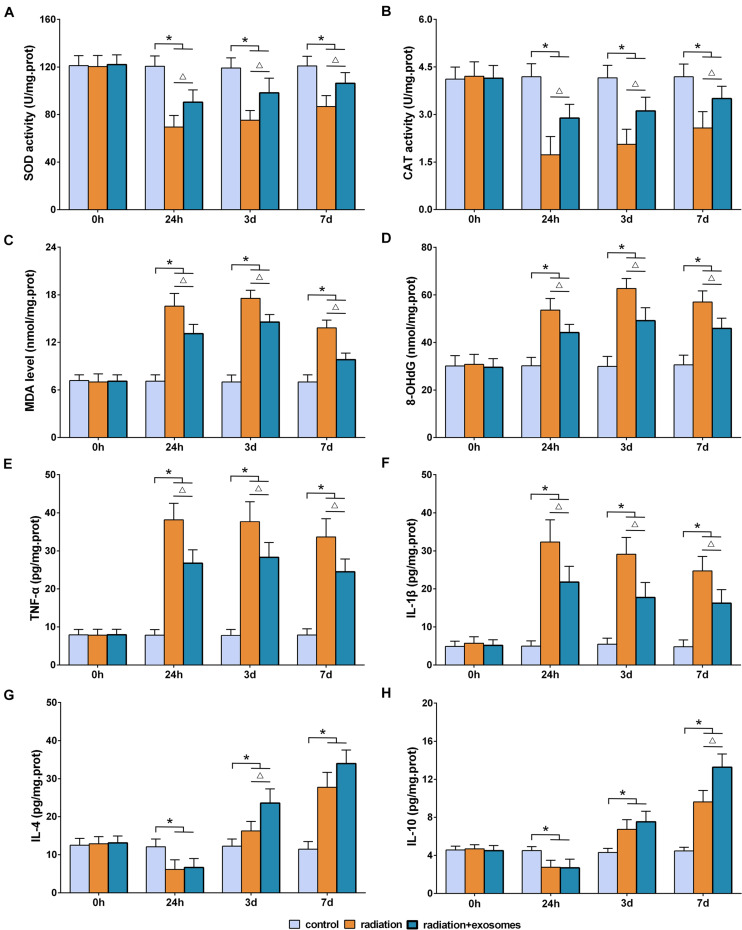
Adipose-derived mesenchymal stem cells alleviated radiation-induced oxidative stress and inflammation in the hippocampus. Antioxidant enzymes SOD **(A)** and CAD **(B)**. Oxidative products MDA **(C)** and 8-OhdG **(D)**. Pro-inflammatory cytokines TNF-α **(E)** and IL-1β **(F)**. Anti-inflammatory cytokines IL-4 **(G)** and IL-10 **(H)**. *p < 0.05, compared with the Ctr; ^△^ *p* < 0.05, compared with the RT. *n* = 6.

The biomarkers of SOD, CAT, MDA, 8-OHdG, TNF-α, IL-1β, IL-4, and IL-10 in the hippocampus were measured preoperation (0 h) and at 24 h, 3 days, and 7 days after irradiation. SOD and CAT are the antioxidant enzymes which can counter the detrimental effects of free oxygen radicals. The results showed that the activities of SOD and CAT in the RT and RT + Exo groups were significantly lower than those in the Ctr group at all time points after irradiation (*p* < 0.05). Statistical analysis showed that the decreases in the SOD and CAT activities in the RT group were more significant than those in the RT + Exo group (*p* < 0.05) (Figures 3A,B).

Malondialdehyde and 8-OHdG are commonly measured indicators of free radical-induced damage. After irradiation, the levels of MDA and 8-OHdG in the RT and RT + Exo groups elevated at 24 h, reached a peak at 3 days, and decreased at 7 days. At all time points after irradiation, the levels of MDA and 8-OHdG in the RT and RT + Exo groups were significantly higher than those in the Ctr group (*p* < 0.05). Statistical analysis found significant differences in the levels of MDA and 8-OHdG between the RT group and the RT + Exo group at 24 h, 3 days, and 7 days after irradiation (*p* < 0.05) (Figures 3C,D).

In the RT and RT + Exo groups, the levels of TNF-α and IL-1β significantly increased at 24 h after irradiation, whereas the levels of IL-4 and IL-10 significantly decreased at 24 h after irradiation. The levels of TNF-α, IL-1β, IL-4, and IL-10 in the RT and RT + Exo groups were significantly higher than those in the Ctr group at 3 and 7 days after irradiation (*p* < 0.05). And the levels of TNF-α and IL-1β in the RT group were significantly higher than those in the RT + Exo group at all time points after irradiation (*p* < 0.05) (Figures 3E,F). Statistical analysis found significant differences in the levels of IL-4 at 3 days after irradiation and the levels of IL-10 at 7 days after irradiation between the RT group and the RT + Exo group (*p* < 0.05) (Figures 3G,H). Here, our results demonstrated that ADMSC-Exos had potent ability to alleviate radiation-induced oxidative stress and inflammation in the hippocampus.

### Adipose-Derived Mesenchymal Stem Cells Inhibited Radiation-Induced Microglial Infiltration and Promoted the Expression of SIRT1 in the Hippocampus

**FIGURE 4:**
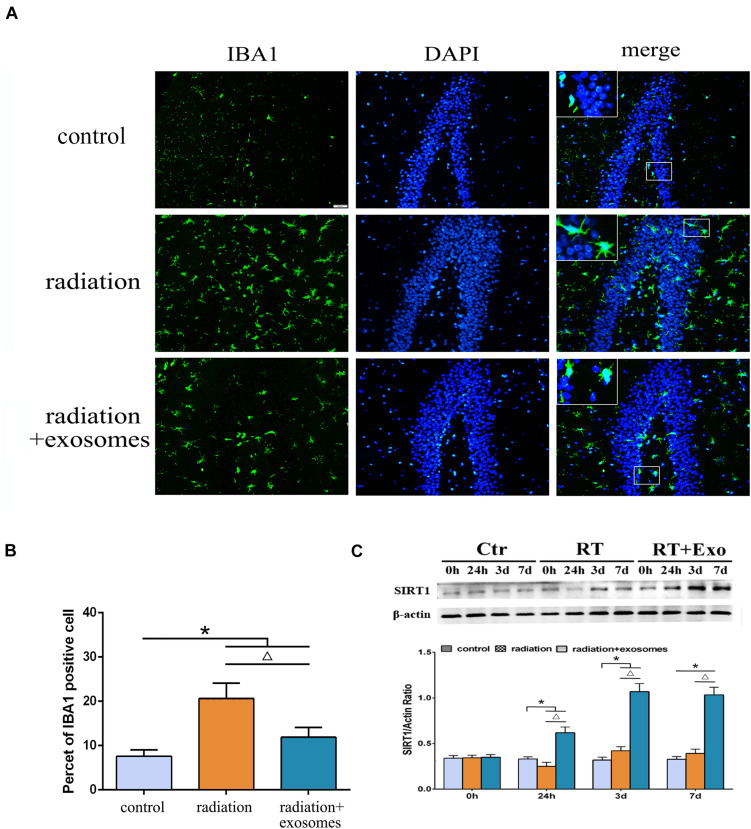
Adipose-derived mesenchymal stem cells inhibited radiation-induced microglial infiltration and promoted the expression of SIRT1 in the hippocampus. **(A)** Microglia were stained by IBA1 (green), the nuclei were stained by DAPI (blue) (scale bar = 50 μm). **(B)** Cell count result of IBA1 positive cell in the hippocampus at 3 days after irradiation (*n* = 6). **(C)** Western blot analysis of SIRT1 expression in the hippocampus after irradiation (*n* = 3) (*p < 0.05, compared with the Ctr; ^△^ *p* < 0.05, compared with the RT).

Immunohistochemistry was performed to observe changes in microglia at 3 days after irradiation. In the RT and RT + Exo groups, the morphological changes of microglia were process retraction and cell body enlargement (Figure 4A). The cell count results showed that there were significantly more IBA1-positive cells in the RT and RT + Exo groups than in the Ctr group (*p* < 0.05), and there were significantly fewer IBA1-positive cells in the RT + Exo group than in the RT group (*p* < 0.05) (Figure 4B).

The protein expression of SIRT1 in the hippocampus was measured preoperation (0 h) and at 24 h, 3 days, and 7 days after irradiation. Western blot analysis showed that in the RT and RT + Exo groups, the protein expression of SIRT1 decreased at 24 h but significantly increased at 3 and 7 days after irradiation. Statistical analysis showed that at 7 days after irradiation, the protein expression of SIRT1 in the RT + Exo group was significantly higher than that in the RT group (*p* < 0.05) (Figure 4C). These results indicated that radiation-induced microglial infiltration associated with the decreased expression of SIRT1, and ADMSC-Exos might inhibit radiation-induced microglial infiltration by promoting SIRT1 expression.

### Adipose-Derived Mesenchymal Stem Cells Alleviated Radiation-Induced Oxidative Stress and Inflammation, and Inhibited the Expression of CD68 in Microglia

**FIGURE 5:**
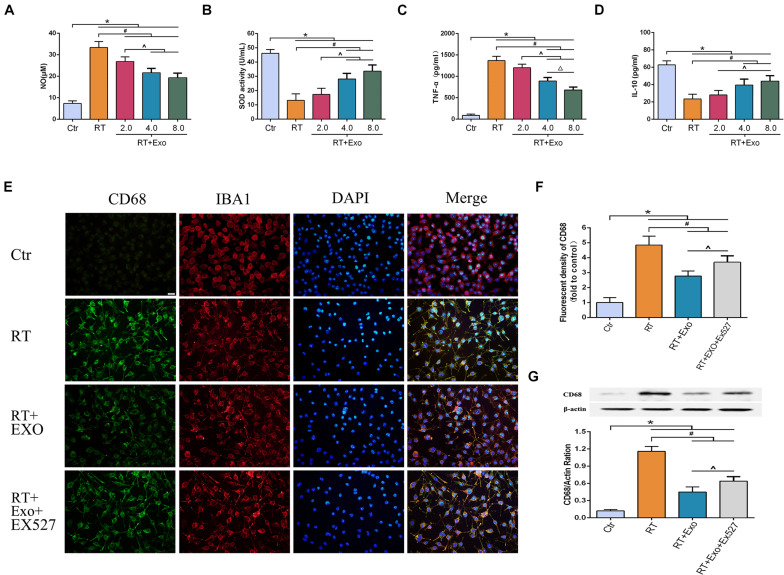
Adipose-derived mesenchymal stem cells alleviated radiation-induced oxidative stress and inflammation, and inhibited the expression of CD68 in microglia. Microglia were pretreated with exosomes (2.0, 4.0, or 8.0 μg) 15 min before irradiation. Expressions of NO **(A)**, SOD **(B)**, TNF-α **(C)**, and IL-10 **(D)** at 24 h after irradiation measured by ELISA (*n* = 6) (*p < 0.05, compared with the Ctr; ^#^*p* < 0.05, compared with the RT; ^*p* < 0.05, compared with the Exo 2.0 μg; ^△^ *p* < 0.05, compared with the Exo 4.0 μg). **(E)** Microglia were stained by CD68 (green) and IBA1 (red), the nuclei were stained by DAPI (blue) (scale bar = 20 μm). EX527 abolished the effect of ADMSC-Exo on the expression of CD68 in microglia. **(F)** Fluorescent density of CD68 at 24 h after irradiation (*n* = 6). **(G)** Expression of CD68 at 24 h after irradiation determined by Western blot (*n* = 3) (*p < 0.05, compared with the Ctr;^ #^*p* < 0.05, compared with the RT;^*p* < 0.05, compared with the Exo).

The activities of SOD and the levels of NO, TNF-α, and IL-10 were measured at 24 h after irradiation. The levels of NO and TNF-α in the RT and RT + Exo groups were significantly higher than those in the Ctr group (*p* < 0.05) (Figures 5A,C), and the activities of SOD and the levels of IL-10 in the RT and RT + Exo groups were significantly lower than those in the Ctr group (*p* < 0.05) (Figures 5B,D). After administration of different concentrations of ADMSC-Exos, the levels of NO and TNF-α substantially decreased. In addition, ADMSC-Exos intervention significantly increased the SOD activities and IL-10 levels. These data revealed a dose-dependent effect, and the optimal concentration of exosomes was confirmed to be 0.4 μg/ul.

CD68 was one of the phenotypic gene markers of activated microglia. The immunofluorescence images revealed that microglia highly expressed CD68 after irradiation (Figure 5E). Results of fluorescent density and Western blotting showed that the expression of CD68 in the Exo group was significantly lower than that in the RT group at 24 h after irradiation (*p* < 0.05). However, the effect of ADMSC-Exos intervention on CD68 expression could be reversed by the SIRT1 inhibitor EX527 (Figures 5F,G). These results further demonstrated that the effect of ADMSC-Exos on microglia was associated with SIRT1 pathway.

### Adipose-Derived Mesenchymal Stem Cells Alleviated Radiation-Induced Oxidative Stress and Inflammation in Microglia via the SIRT1 Pathway

**FIGURE 6:**
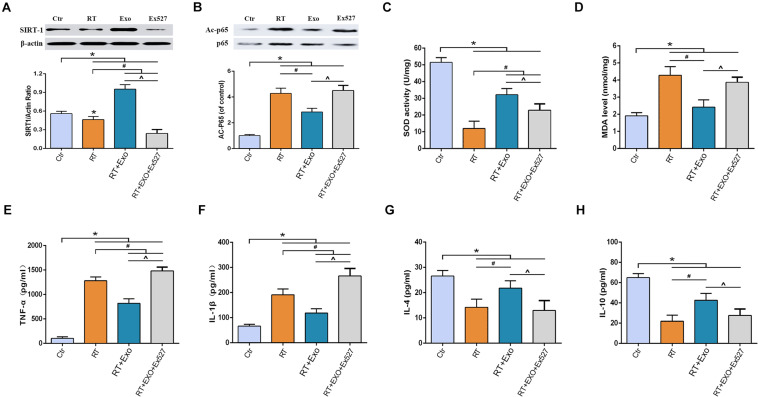
EX527 reversed the effect of ADMSC-Exo on the expressions of SIRT-1 and acetylated p65 in microglia. Expressions of SIRT-1 **(A)** and Acetylated p65 **(B)** at 24 h after irradiation determined by Western blot (*n* = 3). And EX527 abolished the protective effects of ADMSC-Exo on radiation-induced oxidative stress and inflammation in microglia. Expressions of SOD **(C)**, MDA **(D)**, TNF-α **(E)**, IL-1β **(F)**, IL-4 **(G)**, and IL-10 **(H)** at 24 h after irradiation measured by ELISA (*n* = 6) (*p < 0.05, compared with the Ctr;^ #^*p* < 0.05, compared with the RT;^*p* < 0.05, compared with the Exo).

Results of western blotting showed that the expression of SIRT-1 in the RT group was significantly lower than that in the Ctr group, and the expression of acetylated p65 in the RT group was significantly higher than that in the Ctr group (*p* < 0.05). Statistical analysis showed that ADMSC-Exos intervention could significantly increase the expression of SIRT-1 and decrease the expression of acetylated p65, but these effects could be reversed by EX527 (Figures 6A,B).

After irradiation, the activities of SOD and the levels of IL-4 and IL-10 significantly decreased, and the levels of MDA, TNF-α, and IL-1β significantly increased, compared with those in the CTR group (*p* < 0.05). ADMSC-Exos intervention significantly reversed above effects. However, the effects of ADMSC-Exos intervention could be abolished by EX527 (Figures 6C–H). All these results suggested that the exosome-mediated activation of SIRT1 signaling could be abolished by EX527, and the protective effects of ADMSCs-Exos on radiation-induced oxidative stress and inflammation was SIRT1 pathway dependent.

## Discussion

Therapeutic irradiation can cause significant injury to any part of the central or peripheral nervous system. Several studies have shown that stem cell-based interventions can effectively attenuate and repair radiation-associated injuries (Smith and Limoli, 2017; Rodgers and Jadhav, 2018). Here, we demonstrated that ADMSC-Exos exerted protective effects against radiation-induced brain injury by attenuating oxidative stress damage and reducing inflammation and microglial infiltration. Our findings suggest that ADMSC-Exos represent a promising cell-free therapy strategy for radiation-induced brain injury.

A variety of MSCs have shown their potential application prospects in the treatment of radiation-induced injury (Kiang, 2016; Xu T. et al., 2018; Qian and Cen, 2020). Among these cells, MSCs derived from bone marrow, umbilical cord blood, the placenta and adipose tissue are the representative candidates for stem cell therapy (Sousa et al., 2014). However, based on the differentiation potential and immunomodulatory effects of MSCs, bone marrow derived MSCs (BMSCs) and ADMSCs are considered the optimal stem cell source for tissue engineering and regenerative medicine (Heo et al., 2016). However, BMSCs-based therapies are hindered by donor site morbidity, low stem cell concentrations, low available tissue volumes, and compromised cell number and function in elderly patients or those with skeletal diseases, such as osteoporosis. Adipose tissue provides an attractive alternative source of MSCs due to larger available tissue volumes, higher concentrations of stem cells, and reduced donor site morbidity. Moreover, studies have showed that ADMSCs are less influenced by aging and skeletal conditions than BMSCs (Holmes et al., 2021). Therefore, in this study, we chose ADMSCs as the source of exosomes.

Brain injury induced by radiation therapy has traditionally been classified according to the time of onset into acute, early delayed, and late forms (Balentova and Adamkov, 2015). While obvious tissue damage generally occurs after relatively high radiation doses, cognitive impairment can be observed after lower exposures (Tang et al., 2017). The hippocampus is well known to be in charge of study, memory and cognition, so our *in vivo* study was mainly focused on changes in the hippocampus after radiation exposure. The specific mechanisms responsible for cognitive injury are not well understood but may involve changes in the microenvironment, which could be affected by oxidative stress and inflammation. A high rate of oxidative metabolism is a hallmark of the brain. Irradiation could induce the body to generate large amounts of reactive oxygen species (ROS) (Tseng et al., 2014). When ROS generation exceeds the capacity of the cells to protect or repair themselves, excessive ROS could cause cell damage and necrosis. MDA and 8-OHdG are commonly measured indicators of free radical-induced lipid peroxidation and systemic oxidative DNA damage, respectively. The detrimental effects of free oxygen radicals can be countered by the antioxidant enzymes SOD and CAT. In the *in vivo* study, we found that ADMSC-Exos could mitigate the decrease in SOD and CAT activities, as well as attenuate the increase in MDA and 8-OHdG. In addition to causing oxidative stress injury, studies have shown that radiation therapy causes inflammation, which in turn induces neuronal precursor cells in the hippocampus to differentiate into glia instead of neurons (Han et al., 2016; Zhou et al., 2017). This negative effect on neuronal progenitor cells could cause cognitive decline after radiation therapy (Makale et al., 2017; Pulsifer et al., 2018). In the *in vivo* study, we found that the pro-inflammatory cytokines TNF-α and IL-1β significantly increased and the anti-inflammatory cytokines IL-4 and IL-10 significantly decreased in the hippocampus after irradiation, but those changes could be attenuated by ADMSC-Exos. Previous studies have shown that MSC transplantation could attenuate radiation-induced brain injury (Acharya et al., 2015; Liao et al., 2017; Smith and Limoli, 2017), which was similar to the results observed in our study. However, transplantation of stem cells can cause additional physical damage to brain tissue, while intravenous injection of exosomes is relatively safe.

Microglia, the resident immune macrophages of the central nervous system (CNS), are intricately branched and respond rapidly to pathological changes in the brain parenchyma such as excitotoxicity, neurodegenerative insults, ischemia, and direct tissue damage (Ginhoux et al., 2010; Fu et al., 2012). The major findings of recent studies are that microglia can be polarized to become tumor-supportive and immunosuppressive cells by certain tumor-derived soluble factors, thereby promoting tumor maintenance and progression (Graeber et al., 2002; Wu and Watabe, 2017). Moreover, microglia also participate in tumor angiogenesis, metastasis, dormancy, and relapse (Tabouret et al., 2012; Wu and Watabe, 2017). In the present study, radiation-induced microglial infiltration and activation were observed *in vivo* and *in vitro*. It has been proven that after intravenous injection of exosomes through the tail vein, exosomes predominantly distributed in the liver, followed by the brain and then the spleens and lungs (Tian et al., 2021). In the CNS, after intranasal administration of exosomes, exosomes could incorporate robustly into neurons and microglia in rostral regions of the cerebral cortex, and predominantly into neurons in the cortex and the hippocampus at dorsal hippocampal levels. In addition, exosomes were frequently seen adjacent to processes of astrocytes and microglia throughout the frontoparietal cortex and the hippocampus (Long et al., 2017). Our results showed that ADMSC-Exos intervention could significantly reduce the number of activated microglia in the hippocampus and inhibit the expression of CD68 in microglia. Although our results confirmed the regulatory effect of ADMSC-Exos on microglia, the application of exosomes in brain tumor patients receiving radiotherapy should be extremely cautious. Because MSC-derived exosomes (MSC-Exos) are considered a double-edged sword in tumor therapy (Vakhshiteh et al., 2019). Several studies suggest that MSC-Exos perform as mediators in the tumor niche and play several roles in tumorigenesis, angiogenesis, and metastasis (Bruno et al., 2013; Yang et al., 2015; Qi et al., 2017). In contrast, there are other studies supporting the tumor-suppressing effects of MSC-Exos (Bruno et al., 2013; Wu et al., 2013; Takahara et al., 2016). More evidence is needed to evaluate the safety of MSC-Exos in radiotherapy.

SIRT1 is a highly conserved mammalian NAD+-dependent histone deacetylase. In recent years, an increasing number of studies have shown that SIRT1 not only plays a regulatory role in the inflammatory response but also has a close relationship with oxidative stress, glial cell proliferation and tumor recurrence (Singh et al., 2017; Rada et al., 2018; Yan et al., 2019). In the CNS, SIRT-1 plays an important role in promoting neurodevelopment, delaying brain senescence, maintaining homeostasis, and modulating circadian rhythm (Herskovits and Guarente, 2014; Xu J. et al., 2018). Furthermore, a growing number of studies have shown that SIRT-1 exhibits a key role in regulating neuroinflammation via inhibiting NACHT domain-, leucine-rich repeat-, and PYD-containing protein 3 (NLRP3) inflammasome activation, toll-like receptor (TLR) 4 signaling, nuclear factor- (NF-) κB pathway, and IL-1β transcription, which may relate to the modulating of microglial function (Zhang et al., 2019; Zhu et al., 2020). In the *in vivo* study, elevated levels of inflammatory cytokines, decreased expression of SIRT1 and microglial infiltration were observed. Therefore, we hypothesized that SIRT1 may be a key factor in ADMSC-Exo-mediated microglial regulation. In the *in vitro* study, we found that microglia were activated and polarized to the M1 phenotype at the acute phase after irradiation. The ADMSC-Exos intervention could significantly increase the expression of SIRT-1, thereby reducing the acetylation of the NF-κB subunit p65, which ultimately resulted in the reduction of oxidative stress and inflammation. More importantly, the effects of ADMSC-Exos intervention on microglia could be reversed by the SIRT-1 inhibitor EX527. All results verified our hypothesis that SIRT1 was a key factor in ADMSC-Exo-mediated microglial regulation, and further demonstrated that the radioprotective effects of ADMSC-Exos was SIRT-1/NF-κB signaling pathway dependent.

In this presented study, we chose the dose of 30 Gy to establish the rat model of radiation-induced brain injury. This radiation dose can induce changes in rat brains and is the minimum required to produce white matter necrosis without deaths or gross neurologic deficits, and is a well-established animal model for the evaluation of late radiation-induced brain injury (Wang et al., 2009). Lamproglou et al. (1995) reported a radiation-induced (30 Gy) memory deficit at 1 month after irradiation in 4-month-old Wistar rats. Hodges et al. (1998) reported that low dose (20 Gy) irradiation could produce cognitive deficits in rats, but no behavioral changes were observed in the acute phase. Therefore, in this short-term study, we did not assess the effect of ADMSC-Exos on cognition. In addition, it should be noted that our results were obtained in male rats. The study from Hinkle et al. showed that there was a significant basal sex difference in microglial morphology, and irradiation-mediated alterations of microglia and dendritic spine density was sex-specific (Hinkle et al., 2019). The research on radiofrequency radiation found that male rats showed a significant increased, exposure-related DNA damage than female rats, and male mice generally displayed more exposure-related DNA damage than female mice in the hippocampus, cerebellum, and liver (Smith-Roe et al., 2020). Further long-term studies will be required to assess the effects of ADMSC-Exos on radiation-induced cognitive impairment and whether their effects are sex-specific.

In conclusion, we provided direct evidence that ADMSC-Exos exerted protective effects against radiation-induced brain injury by reducing oxidative stress, inflammation and microglial infiltration via activating the SIRT-1 signaling pathway. Compared with other strategies, we believe that the ADMSC-Exo-based approach might be a safer and more promising therapeutic strategy for radiation-induced brain injury. However, the precise mechanism by which ADMSC-Exos alleviate radiation-induced brain injury requires further investigation.

## Data Availability Statement

The original contributions presented in the study are included in the article/supplementary material, further inquiries can be directed to the corresponding authors.

## Ethics Statement

The animal study was reviewed and approved by the Institutional Animal Care and Use Committee of the Air Force Military Medical University.

## Author Contributions

MDL, YSY, and BZ contributed to conception and design of the study. MDL, YSY, and YFY Performed the experiments. JW organized the database. KS and XKY performed the statistical analysis. MDL, GXZ, and JTH wrote the manuscript. All authors contributed to manuscript revision, read, and approved the submitted version.

## Conflict of Interest

The authors declare that the research was conducted in the absence of any commercial or financial relationships that could be construed as a potential conflict of interest.

## Publisher’s Note

All claims expressed in this article are solely those of the authors and do not necessarily represent those of their affiliated organizations, or those of the publisher, the editors and the reviewers. Any product that may be evaluated in this article, or claim that may be made by its manufacturer, is not guaranteed or endorsed by the publisher.
